# *In situ* modified nanocellulose/alginate hydrogel composite beads for purifying mining effluents†

**DOI:** 10.1039/d3na00531c

**Published:** 2023-10-02

**Authors:** Dimitrios Georgouvelas, Hani Nasser Abdelhamid, Ulrica Edlund, Aji P. Mathew

**Affiliations:** a Division of Materials and Environmental Chemistry, Stockholm University Svante Arrhenius väg 16C Stockholm SE-10691 Sweden aji.mathew@mmk.su.se +468161256; b Advanced Multifunctional Materials Laboratory, Department of Chemistry, Faculty of Science, Assiut University Assiut 71515 Egypt; c Department of Fibre and Polymer Technology, School of Engineering Sciences in Chemistry, Biotechnology, and Health, KTH Royal Institute of Technology Teknikringen 56 Stockholm SE-10044 Sweden

## Abstract

Biobased adsorbents and membranes offer advantages related to resource efficiency, safety, and fast kinetics but have challenges related to their reusability and water flux. Nanocellulose/alginate composite hydrogel beads were successfully prepared with a diameter of about 3–4 mm and porosity as high as 99%. The beads were further modified with *in situ* TEMPO-mediated oxidation to functionalize the hydroxyl groups of cellulose and facilitate the removal of cationic pollutants from aqueous samples at low pressure, driven by electrostatic interactions. The increased number of carboxyl groups in the bead matrix improved the removal efficiency of the adsorbent without compromising the water throughput rate; being as high as 17 000 L h^−1^ m^−2^ bar^−1^. The absorptivity of the beads was evaluated with UV-vis for the removal of the dye Methylene Blue (91% removal) from spiked water and energy dispersive X-ray spectroscopy (EDS) and X-ray photoelectron spectroscopy (XPS) elemental analyses for the removal of Cd^2+^ from industrial mining effluents. The modified beads showed a 3-fold increase in ion adsorption and pose as excellent candidates for the manufacturing of three-dimensional (3-D) column filters for large-volume, high flux water treatment under atmospheric pressure.

## Abstract 2

Biobased adsorbent beads with anionic charge used in three-dimensional (3-D) column filters for large-volume, high flux water treatment under atmospheric pressure.

## Graphical abstract

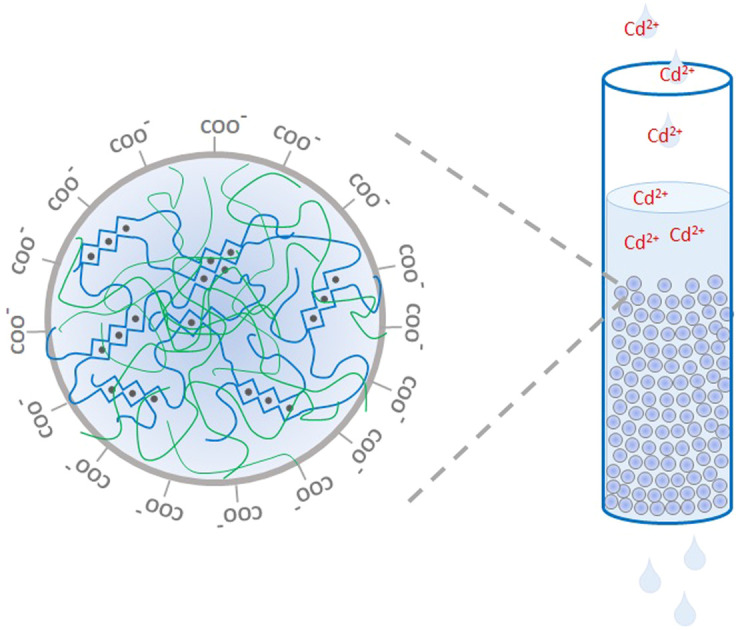

## Introduction

Industrial wastewater treatment before its discharge is crucial to minimize the contamination of water streams with hazardous or potentially hazardous substances, for instance, heavy metal ions and dyes. Literature reports show the successful use of inorganic materials *e.g.*, carbon, metal oxide, and their combinations produce adsorbents and membranes with catalytic properties or magnetic properties for the removal of metal ions and dyes.^1–5^

Cellulose and nanocellulose (either cellulose nanocrystals CNC or cellulose nanofibril (CNF)) have emerged in recent years as versatile biobased choices for water treatment^6–11^ thanks to their meritorious properties, such as low environmental impact, high natural abundancy, and versatile surface chemistry which allows for functionalization.^6,7^ Cellulose offers an abundance of hydroxyl groups (–OH) which are susceptible to modification. An efficient method to introduce carboxyl groups (–COO–) and increase the affinity of cellulose towards cationic species is by selectively oxidizing the primary alcohol of its structure *via* 2,2,6,6-tetramethylpiperidine-1-oxyl (TEMPO) mediated oxidation^12^ Currently, TEMPO oxidation constitutes the most used pretreatment method (before mechanical disintegration of cellulose pulps) for the preparation of highly charged CNF (often denoted as TEMPO-CNF or TO-CNF) and has been reported as a successful *in situ* modification method of cellulosic membranes.^13^ Despite the broad use of cellulose membranes in water purification, there are certain limitations in their capabilities.

The typically dense structure of the bodies of membranes leads to a high-pressure drop when used in dead-end flow and cross-flow. Pressure drop is defined as the pressure difference between the inlet and the outlet of a filter and it can result in the movement or even fracture of the filter. One way to reduce the pressure drop is to increase the permeability of a membrane either by reducing its thickness or increasing its porosity. However, both approaches involve a tradeoff between the mechanical properties and the efficiency of the filter. A less dense membrane matrix entails that a smaller amount of material is used and fewer functional groups are present to facilitate the adsorption. To overcome these drawbacks, 3-D adsorbents have been developed, for instance in the form of spherically shaped and porous aerogel and hydrogel particles.^14–17^ Such particles were successfully prepared from several different hybrid materials, including graphene/cellulose, poly(sodium acrylate)/cellulose, and starch mixtures, and proven efficient in water purification.^18–20^ The advantages of these adsorbents, often denoted as aerogel or hydrogel beads, are that they offer a higher number of functional groups per area compared to membranes, can be easily removed after water treatment, and can be packed in cylindrical configurations for the manufacturing of filters that resemble the ion exchange resins and allow high water permeance.

**Fig. 1:**
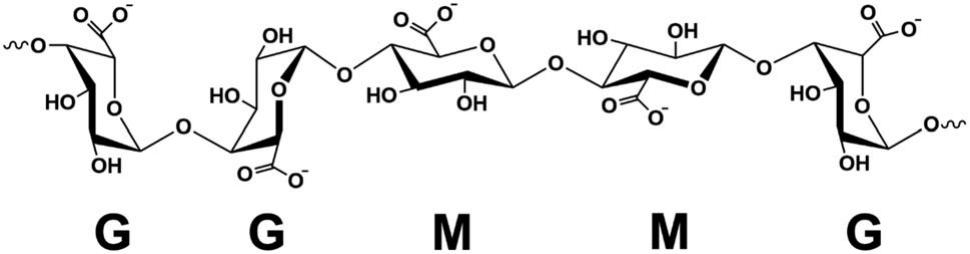
Schematic representation of an alginate structural motif.

A facile method to prepare highly porous fully biobased beads is with the use of alginate/cellulose hydrogel composites.^21–24^ Alginate or alginic acid (commercially available as a sodium salt (SA)) is a linear polysaccharide extracted from brown algae.^21^ It consists of blocks of (1,4)-linked β-d-mannuronate (M) and α-l-guluronate (G) residues and as a copolymer, it is composed of either consecutive G or M units, for instance, GGGG or MMMM, or alternating M and G units, for instance, GMGMGM. Fig. 1 depicts the GGMMG configuration, as an example of an alginate structural motif.

Due to its structure, alginate forms hydrogels upon interaction with ionic cross-linking agents such as divalent cations. When aqueous alginate solutions are poured dropwise into CaCl_2_ solutions they instantly form spherical-shaped hydrogels because of the ionic-crosslinking of the G units of adjacent polymer chains, forming a so-called egg-box structure.^6^ Notably, it is believed that only the G units bind to Ca^2+^ because of their higher degree of coordination.^21^

The carboxyl and hydroxyl groups of alginate and cellulose make cellulose/alginate composites highly promising for use in water treatment applications for the removal of cationic pollutants. Stand-alone alginate^25,26^ as well as cellulose/alginate beads^27,28^ have already been reported for heavy metal ion removal.

We aimed to investigate the effect of *in situ* TEMPO oxidation on nanocellulose/alginate composite beads to control and enhance the performance which has not explored to date. We hypothesize that by oxidizing the available hydroxyl groups of cellulose, the amount of carboxyl groups would increase and thereby improve the removal capacity of the beads. For this purpose, commercial-grade defibrillated cellulose and SA were used for the preparation of beads which were then modified *in situ* by TEMPO-mediated oxidation. The effect of this modification was monitored by comparing the adsorption capacity of the pristine hydrogel beads (denoted as C-SA) with that of TEMPO-oxidized beads (denoted as TO-C-SA) toward Methylene Blue (MB, a cationic model dye) and metal ion (Cd^2+^ from mining effluent), respectively.

## Experimental

### Materials and methods

Commercial grade defibrillated cellulose with micro- and nanosized fibrils (Exilva, 10.1% (w/w)) was supplied by Borregaard AB (Sarpsborg, Norway). Alginic acid sodium salt (SA), calcium chloride, sodium bromide, cadmium nitrate tetrahydrate, ethanol (EtOH 95%), and Methylene Blue (MB) were purchased from Sigma-Aldrich. Poly(diallyl dimethyl ammonium chloride) (PDADMAC, 20% in H_2_O) was used as received from Sigma-Aldrich. Sodium hypochlorite and hydrochloric acid were purchased from VWR. 2,2,6,6-Tetramethylpiperidine-1-oxyl (TEMPO) was purchased from TCI. All chemicals were used as received. Samples from Vormbäcken in Sweden, a water recipient of mining operation effluents, were kindly provided by Boliden.

#### Composite hydrogel and beads preparation

SA (1 g) was vigorously mixed in 100 mL distilled H_2_O at 50 °C until a transparent solution was obtained. The SA solution was then mixed with a 1% (w/w) cellulose dispersion in a 1 : 1 ratio for 1 h. The mixture was then collected with a syringe and was poured dropwise into a CaCl_2_ (0.7 M) solution, through a 2 mm needle (Sterican, Braun) using a syringe pump at a rate of 0.5 mL min^−1^ (A video of the process is shown in the ESI,† S1). Despite their instantaneous formation, the beads were left in the CaCl_2_ solution for 30 more min for stabilization. Finally, the beads were collected, washed with H_2_O, and stored in H_2_O in the fridge. Before every use hereafter, unless otherwise noted, the beads were placed on dry paper for a few seconds to remove the excess absorbed water and have better control over their amount.

### *In situ* TEMPO oxidation of beads

TEMPO (0.03 g, 0.2 mM) was dispersed and 0.5 g NaBr (5 mM) was dissolved in 100 mL H_2_O. Then, 4.5 mL NaClO (aq) (10% v/v, 6 mM) was added and the pH of the reaction mixture was adjusted to 10 with dilute HCl (aq). Then, 10 g of partially dried hydrogel beads were added to the reaction mixture for 5 min. The beads were removed and transferred to an EtOH : H_2_O (1 : 4 ratio) mixture to quench the reaction for 1 min. Finally, the beads were washed with H_2_O and stored in H_2_O in the fridge until further use.

### Characterization of surface chemistry of the beads

Fourier transfer infrared spectroscopy (FTIR) spectra of lyophilized beads were recorded in the range of 400–4000 cm^−1^, through 64 scans, and a resolution of 4 cm^−1^ with an ATR-FTIR spectrometer (670-IR, Varian). All obtained spectra were baseline-corrected.

### Determination of surface charge density

Beads (1 g) were dispersed in 100 mL Milli-Q H_2_O. The mixture was vigorously mixed with UltraTurrax (IKA) at 15 000 rpm until a homogeneous dispersion was obtained. Aliquots (1 mL) of the dispersions (pristine and *in situ* oxidized beads, respectively) were diluted with Milli-Q H_2_O to 100 mL and titrated with PDADMAC using a Stabino (ParticleMetrix) system. The charge density of PDADMAC is 0.307 μeq mL^−1^. The measurements were replicated three times for each sample and the surface charge densities were calculated according to eqn (1):1
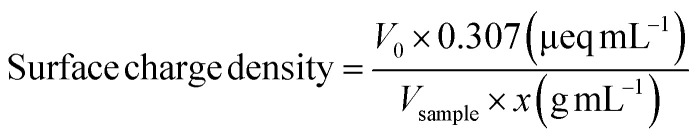
where *V*_0_ is the volume of titrant consumed to neutralize the sample, *V*_sample_ is the volume of the sample, and *x* is the concentration of the sample.

### Morphology of the beads

Images of the surface of the prepared beads were recorded with a USB optical microscope (AM73915MZT, Dino-Lite Digital Microscope). Moreover, the morphology of the surface and the cross-section of lyophilized beads were observed using a scanning electron microscope (SEM, TEM3000, Hitachi) with an acceleration voltage of 5 kV.

### Porosity

The porosity of the beads was calculated by the gravimetric method using eqn (2).^23^2
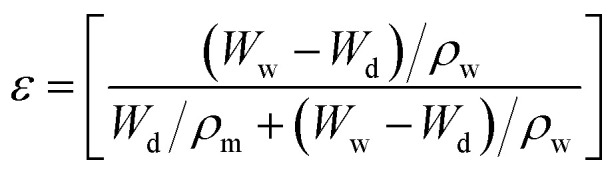
where *W*_w_ and *W*_d_ are the weights in grams of the wet and dry beads, respectively, and *ρ*_m_ and *ρ*_w_ are the densities of the bead matrix and water, respectively, in g cm^−3^.

### Determination of MB removal

Aqueous solutions of MB (5 mg L^−1^) were prepared. Beads (1 g) were immersed in 100 mL dye solutions and aliquots were removed at specific times (after 1, 5, 10, 30, 60, 120, 240, 300, and 720 min). The aliquots were then analyzed with UV-vis spectroscopy (Agilent Cary 5000 UV/vis/NIR, Agilent) in the spectral range 200–800 nm and the maximum absorbance at 665 nm (corresponding to MB) was recorded.

### Determination of Cd^2+^ removal

Aqueous solutions of Cd^2+^ (400 mg L^−1^) were prepared. Beads (1 g) were immersed in 100 mL Cd^2+^ solutions and left for 1 h. The beads were then collected and dried at 40 °C under vacuum. Elemental analysis of the beads was performed with energy-dispersive X-ray spectroscopy (EDS) using a scanning electron microscope equipped with an X-ray detector (TEM3000, Hitachi), at an acceleration voltage of 15 kV.

Similarly, X-ray photoelectron spectroscopy (XPS) analysis of beads after interaction with Cd^2+^ solution was performed *via* Thermo Fisher (K-Alpha) with micro-focused Al Kα radiation (energy of 1486.6 eV). A carbon peak at a binding energy of 284.2 eV was used as a reference. For these experiments, samples from effluents of a mining industry were used to study the selectivity of the beads towards Cd^2+^.

### Flux measurement

The time needed for 100 mL H_2_O to pass through a chromatographic column with a beaded rim (Lenz, 30 mm diameter) was recorded. The measurement was repeated with the use of commercial filter paper (average pore size 6 μm, Munktell, Grade 3) and 1, 5, and 10 g of the beads. The reported values are averages of 5 replicates.

## Results and discussion

### Surface chemistry of the beads

**Fig. 2:**
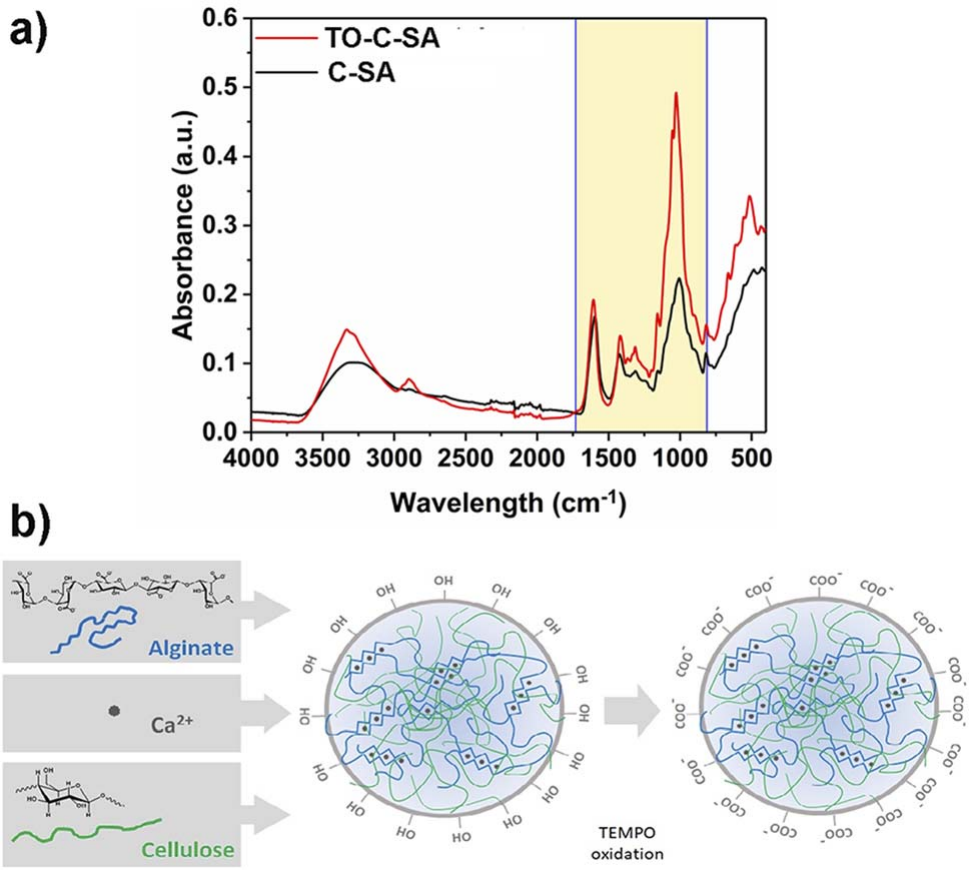
(a) FT-IR spectra and (b) cellulose-SA bead formation *via* Ca^2+^ complexation followed by surface modification by TEMPO oxidation.

Functional groups of the prepared beads before and after the *in situ* modification were characterized with FT-IR (Fig. 2a). The recorded spectra show absorption peaks at 1606, 1420, and 1029 cm^−1^ which correspond to stretching vibrations of – –COO^−^ (asymmetric), –COO^−^ (symmetric), and C–O bonds, respectively.^24^ The increase in the intensity of the peaks of TO-C-SA compared to C-SA is attributed to the increase of carboxyl groups after oxidation.

The increase in the number of carboxyl groups in the composition of the beads after oxidation is also supported by an increase in surface charge density from 291 ± 51 mmol kg^−1^ for the C-SA to 477 ± 47 mmol kg^−1^ for TO-C-SA. An earlier report from our group on *in situ* TEMPO oxidation of cellulose membranes (sludge-CNF/CNC_BE_ membranes, “BE” herein stands for “bioethanol” because this CNC was produced from the residue of bioethanol production), showed an increase in acidic group content from ∼7 to ∼42 mmol kg^−1^, corresponding to a significant 6-fold charge increase.^13^ In the current case, the charge density of the beads after modification is significantly higher (almost 10 times), however, the increase between modified and unmodified beads showed only a two-fold increase which can be attributed to the abundance of carboxyl groups in the alginate phase used for beads processing. The concept of bead formation by nanocellulose and alginate by Ca^2+^ crosslinking and subsequent TEMPO oxidation is schematically shown in Fig. 2b.

### Morphology of the beads

**Fig. 3:**
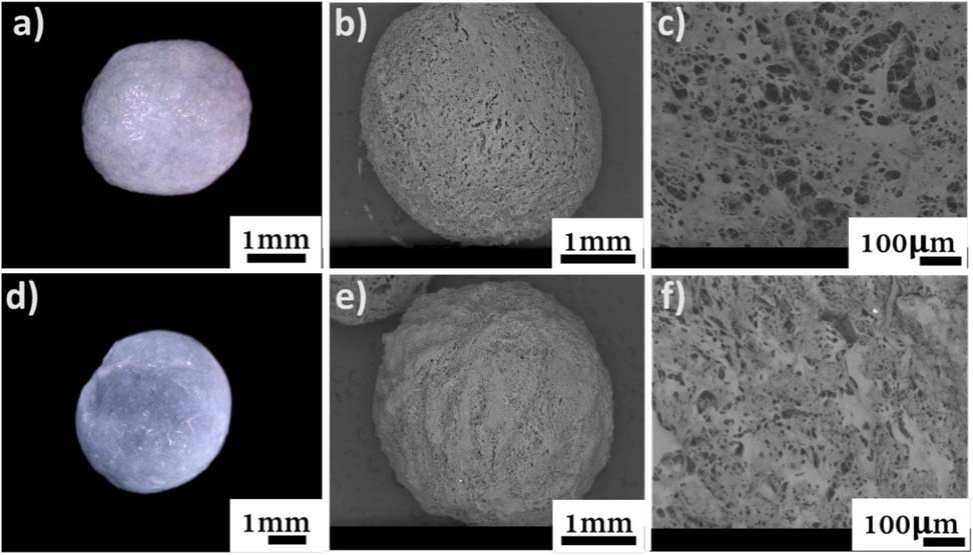
Top (a) digital micrograph, SEM micrographs of (b) surface, and (c) cross-section of C-SA. Bottom, (d) digital micrograph, SEM micrographs of (e) surface, and (f) cross-section of TO-C-SA.

The morphology of the beads was monitored with a digital optical microscope, as well as SEM imaging for their surface and cross-section (Fig. 3).

From the obtained micrographs, there is no significant change in the morphology of the beads after *in situ* oxidation. The average dry weight of the TO-C-SA beads was about half of the C-SA beads while their average wet weight was almost identical. This is probably attributable to the fact that the modified beads are more hydrophilic and, therefore, absorb more H_2_O.^12^ TO-C-SA contains more carboxyl groups than C-SA, explaining the difference in hydrophilicity. Nevertheless, the differences in average dry and wet weights did not significantly affect the porosity of the beads before and after the modification (Table 1).

**Table 1:** Morphological characteristics of C-SA and TO-C-SA beads

	C-SA	TO-C-SA
Average diameter (mm)	3.89 ± 0.25	3.14 ± 0.25
Average wet weight (mg)	63.1	62.3
Average dry weight (mg)	0.78	0.40
Porosity (%)	99.2	99.6

### MB removal

**Fig. 4:**
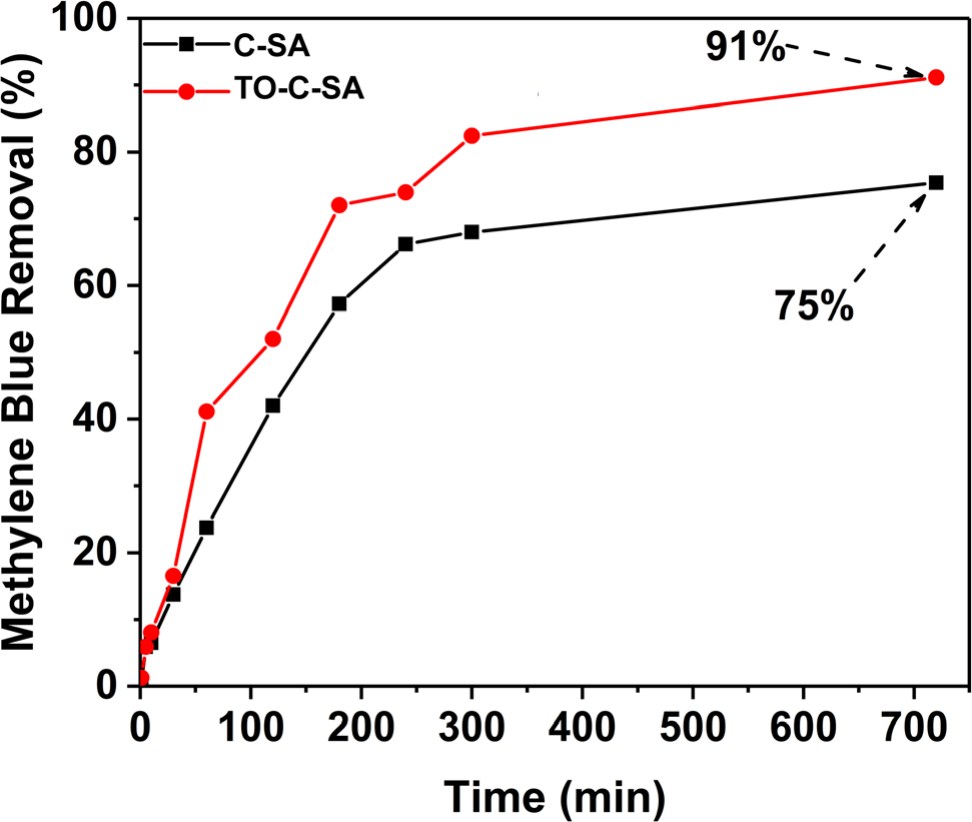
MB removal efficiency for C-SA and TO-C-SA beads.

The effect of the modification on the adsorptivity of the beads toward cationic dyes was monitored with MB, which is positively charged at neutral pH.^29^ The obtained data from UV-vis measurements showed that the pristine beads have an adequate removal efficiency of 75% after 12 h (for initial MB concentration). *In situ* oxidation of the beads increased the total MB removal efficiency, reaching 91% after 12 h (Fig. 4). Adsorption of positively charged dyes shows a systematic increase with carboxyl group content indicating that the adsorption process is primarily driven by electrostatic interactions.

A summary of beads used for dye adsorption is tabulated in Table 2. Chitosan (CTS) beads with montmorillonite (MT) were investigated for methyl green (MG) adsorption from aqueous solutions.^30^ After optimizing many parameters, the beads offered efficiency of 99%. Alginate beads containing polyamidoamine/halloysite nanotubes (Alg/Hal_PAMAM beads) was also reported for MG adsorption.^31^ These composite beads exhibit good adsorption efficiencies. However, it contains inorganic clays *e.g.* montmorillonite or halloysite that can be a source of contamination for long-term exposure. Our beads are pure organic biopolymers without any inorganic materials with good efficiency. It should be also notted that most of these efficiencies are based on the biopolymer of the beads *e.g.*, chitosan.^31^ Cellulose-based beads are economically cheaper than chitosan based beads.^32^ Copper ions were suggested to replace Ca^2+^ for the formation of Graphene oxide (GO)/SA/Carrageenan (GO/Alg-Car) beads (Ala : Car : GO = 2 : 2 : 1).^33^ The use of Ca^2+^ is environmentally benign compared to Cu^2+^ ions that can be released into the aqueoutic system causing secondary pollution. SA beads of *meso*-tetrakis(2,4,6-trimethylphenyl)porphyrinto) zinc(ii) complex (Zn(TMP)) containing 3% of SA was reported for MB adsorption.^34^ TO-C-SA beads exhibit comparable adsorption efficiency with a suitable equilibrium time to reach the steady state (Table 2).

**Table 2:** A comparison among different beads used for dye adsorption

Beads	Dye	Efficiency (%)	Conditions	Time	Ref.
CTS/MT	MG	99%	Beads 5 mg, 500 mg g^−1^ MG, pH 6.0	24 h	30
Alg/Hal_PAMAM	MG	97	Beads 25 mg; 200 mg L^−1^ MG, pH 7	24 h	31
GO/Alg-Car	MB	90	Beads 20 mg, 60 mg L^−1^ MB, pH 9.2	5 h	33
Zn(TMP)/Alg	MB	40	Beads 30 mg, 30 mg L^−1^ MB, pH 6	1.5	34
TO-C-SA	MB	91	Beads 1 g, 5 mg L^−1^, pH 7	12 h	Here

### Cd^2+^ removal

The adsorption capacity of the prepared beads towards Cd^2+^ was studied with EDS and XPS elemental analyses. For the former, spiked Cd^2+^ aqueous samples were used while for the latter, samples from a water recipient of mining discharge were used in an attempt to monitor the selectivity of the beads towards Cd^2+^.

EDS elemental analysis of the beads after immersion in aqueous Cd^2+^ samples showed an increase in Cd^2+^ atomic percentage (at%) from 2.53 to 6.98 (Table 3).

**Table 3:** The atomic percentage of Cd^2+^ and Ca^2+^ in C-SA and TO-C-SA beads

	Cd^2+^ (at%)	Ca^2+^ (at%)
C-SA	2.53	0.44
TO-C-SA	6.98	2.09

This almost 3-fold increase in Cd^2+^ atomic percentage can be attributed to the carboxyl groups generated in the *in situ* oxidation of the beads. In addition, an increase in the atomic percentage of Ca^2+^ can be observed.

**Fig. 5:**
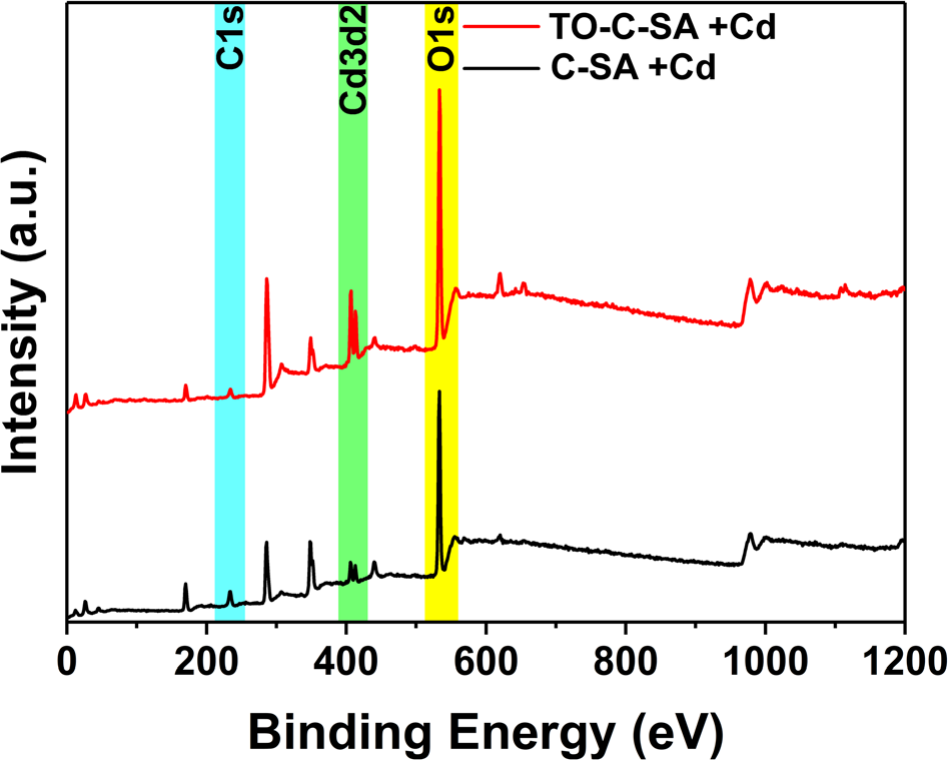
XPS survey of C-SA and TO-C-SA beads after Cd^2+^ adsorption.

The at% of adsorbed Cd^2+^ on the surface of the beads was estimated from the XPS elemental survey. The data indicate an improvement in ion adsorption; the at% of Cd^2+^ increased from 0.84 for the pristine beads to 1.76 for the modified ones (Fig. 5). This again supports the concept of ion adsorption driven by electrostatic interactions. Sehaqui *et al.*^35^ studied the adsorption of divalent metal *e.g.*, Cu^2+^ onto oxidized cellulose and showed that the adsorption increases inearly with carboxylate content with maximum adsorption capacities of 55 mg g^−1^ at neutral pH. It was established that Cu^2+^ ions are adsorbed onto the TCNF *via* electrostatic interactions involving the carboxyl groups on the oxidized cellulose fiber surface.^36^ The proton of the carboxyl group is exchanged with a metal ion during the adsorption process. Although the obtained data from EDS and XPS follow the same trend, there are significant differences in at% values. XPS is a surface analysis technique using a lower energy X-ray beam compared to EDS. In XPS, the beam penetration depth is about 10 nm while EDS assesses the entire bulk. In addition, the XPS measurements were performed on beads that were used to treat samples of actual effluents from mining. The presence of a plethora of charged contaminants may have affected the interaction between the beads and Cd^2+^.

**Fig. 6:**
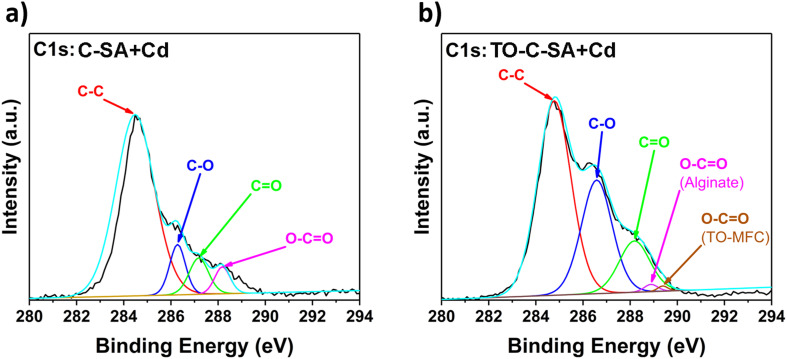
C 1s high-resolution XPS spectra of (a) C-SA and (b) TO-C-SA beads after Cd^2+^ adsorption.

The deconvolution of the high-resolution C 1s spectrum of C-SA beads after Cd^2+^ adsorption reveals distinct peaks at binding energies (BE) of 284.4, 286.6, 287.2, and 288.2 eV which correspond to C–C, C–O, C=O, and O–C=O, respectively (Fig. 6a). Similarly, the deconvolution of the C 1s spectrum of TO-C-SA beads after adsorption shows peaks at BE of 284.8, 286.7, 288.2, 288.9, and 289.4 eV (Fig. 6b). The first three peaks are assigned to C–C, C–O, and C=O, respectively. The peaks at 288.9 and 289.4 eV correspond to the carboxyl groups (O–C=O) for alginate (G or M) and TEMPO-oxidized cellulose (O–C=O), respectively.^37^

**Fig. 7:**
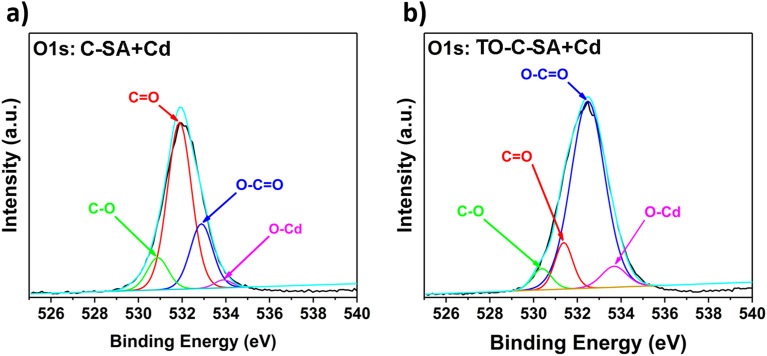
O 1s high-resolution XPS spectra of (a) C-SA and (b) TO-C-SA beads after Cd^2+^ adsorption.

The deconvolution of the high-resolution O 1s spectrum of C-SA beads after Cd^2+^ adsorption reveals peaks at BE of 530.9, 531.9, 532.9, and 533.9 eV, which correspond to C–O, C=O, O–C=O, and O–Cd, respectively (Fig. 7a). From the deconvolution of the O 1s spectrum of TO-C-SA beads after adsorption, peaks at BE of 530.4, 531.4, 532.5, and 533.9 eV are revealed (Fig. 7b). The peaks correspond to the same type of O as in C-SA; however, the intensity of the peak that corresponds to C=O increases, presumably due to the contribution of the carboxyl O atoms that are introduced with the TEMPO oxidation.^38,39^

**Fig. 8:**
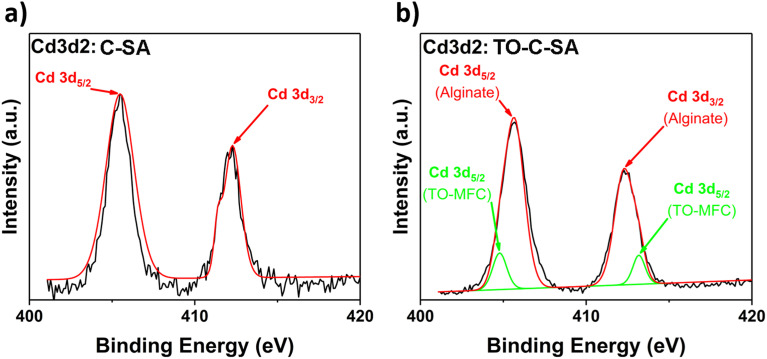
Cd 3d high-resolution XPS spectra of (a) C-SA and (b) TO-C-SA beads after Cd^2+^ adsorption.

Finally, the deconvolution of the high-resolution spectrum of Cd 3d2 of C-SA beads after Cd^2+^ adsorption reveals two distinct peaks at BE of 405.5 and 412.3 eV, corresponding to Cd 3d_5/2_ and Cd 3d_3/2_ types of Cd, respectively (Fig. 8a). Cd^2+^ is adsorbed on the surface of the beads due to interactions with the O atoms of the carbonyl groups of alginate. However, the deconvolution of the Cd 3d2 spectrum of the TO-C-SA beads reveals two additional lower-intensity peaks at 404.8 and 413.2 eV (Fig. 8b). These two peaks presumably correspond to Cd^2+^ ions that are adsorbed on the surface of the beads due to interactions with the carboxyl O atoms that are introduced with the TEMPO oxidation. Binding energies of approximately 7 eV of Cd 3d_5/2_ and Cd 3d_3/2_ are characteristic of the two states of Cd^2+^.^8^

### Loose-fill column filters

**Fig. 9:**
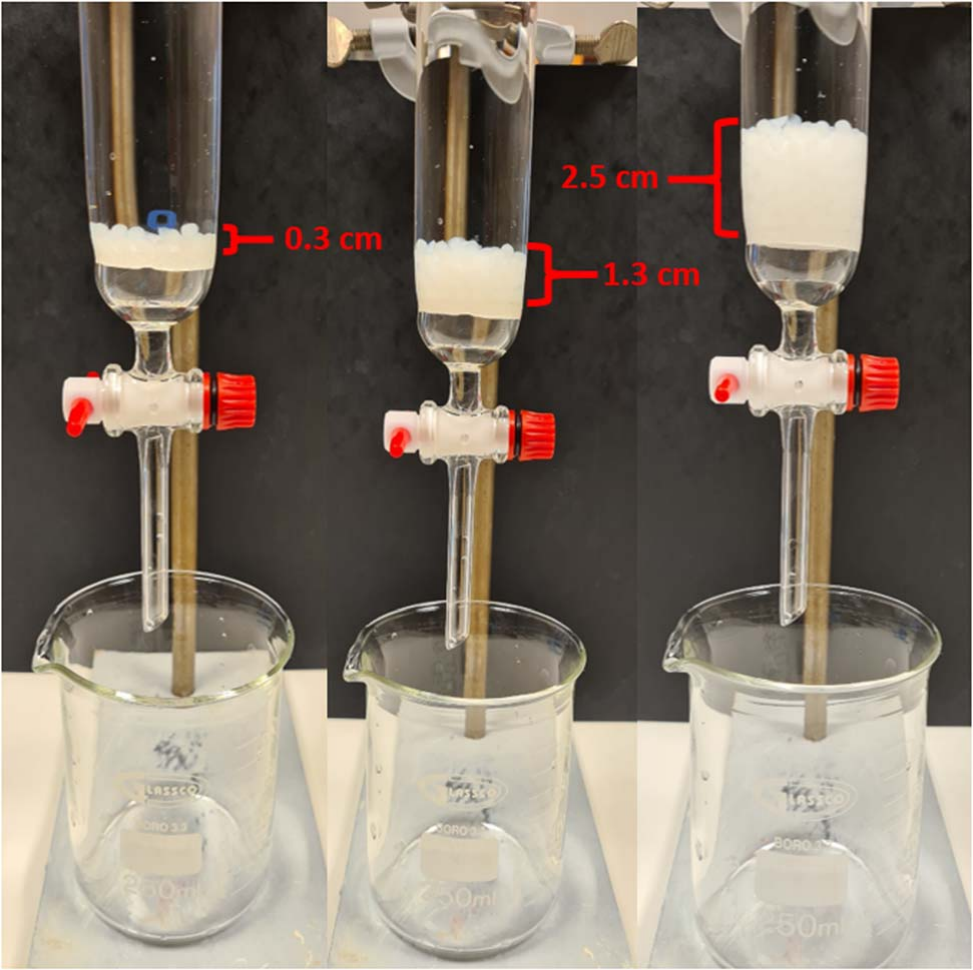
Illustration of loose-fill column filter setup for TO-C-SA beads. 1, 5 and 10 g of beads provided 0.3 cm, 1.3 cm, and 2.5 cm of beaded column respectively.

To evaluate the potential to use the beads in column filters and to compare the effect on water flux, the required time for 100 mL H_2_O to pass through a chromatographic column with a beaded rim under the effect of atmospheric pressure was measured. As a reference, the dewatering time without the use of any adsorbent was measured to be 28.7 ± 2.3 s (flux 177 371 L h^−1^ m^−2^ bar^−1^). The dewatering time increased to 46.3 ± 2.6 s with the use of commercial-grade filter paper. The required dewatering time was 28.8 ± 2.2, 30.5 ± 3.1, and 44.2 ± 3.6 s when packing the column with 1, 5, and 10 g of C-SA beads, respectively (the corresponding flux was calculated to be 16 720, 17 724, 11 541 L h^−1^ m^−2^ bar^−1^) The required dewatering time was identical when instead filling the column with TO-C-SA beads was 28.6 ± 1.1, 30.4 ± 0.7, and 44.0 ± 2.5 s for 1, 5, and 10 g of beads, respectively (the experimental set up is shown in Fig. 9). The obtained dewatering times clearly show that a single layer of beads with 1 g of beads did not affect the water flow. An amount of 10 g of beads, which formed a stack of layers of approximately 2.5 cm, showed better water flux than commercial filter paper. Additionally, the average dewatering times for TO-C-SA and C-SA beads are the same which indicates that, in this range, the functionalization does not affect the water flux. Furthermore, the water flux was found to be higher than that reported for cellulose-based layered membrane systems in previous studies from our group which fluctuated from 3417 to 14 742 L h^−1^ m^−2^ bar^−1^.^13,40–43^

## Conclusions

We devised a facile method of preparing nanocellulose/alginate composite beads with increased adsorption performance derived from the *in situ* TEMPO oxidation of the hydroxyl groups of cellulose. Oxidation of the beads increased the number of carboxyl groups which can facilitate the removal of cationic impurities from aqueous samples. The enhancement of removal efficiency of the cationic dye MB and Cd^2+^ was confirmed with UV-vis measurements (for MB), EDS, and XPS (for Cd^2+^). Interestingly, the EDS elemental analysis of the beads showed an almost 3-fold improvement in Cd^2+^ removal after the *in situ* oxidation. Furthermore, the obtained water flux data indicates that the prepared beads can be used for the manufacturing of column filters. This approach offers filters with a higher amount of adsorbent and better water permeability than 2D membrane filters. Overall, the abundance of the component feedstock, as well as the ease and efficiency of the modification route indicate that the nanocomposite beads are promising candidates for larger production and manufacturing of column filters for water purification. Nevertheless, how the mechanical properties of the beads were affected by the oxidation and how the presence of several charged contaminants in the same sample affects the adsorption performance of the beads will be a subject of future investigation. The long-term stability and recyclability of the beads will also be a topic of further investigation.

## Author contributions

D. G. methodology, data collection, investigation, writing-original draft, D. G.; investigation, writing-review and editing; H. N. A., methodology, data collection, investigation, writing-original draft, D. G.; investigation, writing-review, and editing; A. P. M., conceptualization, supervision, writing-review and editing; U. E., supervision, writing-review and editing. All authors have read and agreed to the published version of the manuscript.

## Conflicts of interest

There are no conflicts to declare.

## Supplementary Material

NA-005-D3NA00531C-s001
